# Nuclear localization of Beclin 1 promotes radiation-induced DNA damage repair independent of autophagy

**DOI:** 10.1038/srep45385

**Published:** 2017-03-27

**Authors:** Fei Xu, Yixuan Fang, Lili Yan, Lan Xu, Suping Zhang, Yan Cao, Li Xu, Xiaoying Zhang, Jialing Xie, Gaoyue Jiang, Chaorong Ge, Ni An, Daohong Zhou, Na Yuan, Jianrong Wang

**Affiliations:** 1 Hematology Center of Cyrus Tang Medical Institute, Collaborative Innovation Center of Hematology, Soochow University School of Medicine, Suzhou 215123, China; 2 Jiangsu Institute of Hematology, Jiangsu Key Laboratory for Stem Cell Research, The First Affiliated Hospital, Soochow University School of Medicine, Suzhou 215123, China; 3 Division of Radiation Health, Department of Pharmaceutical Sciences, University of Arkansas Medical Sciences, Little Rock, Arkansas 72205, USA

## Abstract

Beclin 1 is a well-established core mammalian autophagy protein that is embryonically indispensable and has been presumed to suppress oncogenesis via an autophagy-mediated mechanism. Here, we show that Beclin 1 is a prenatal primary cytoplasmic protein but rapidly relocated into the nucleus during postnatal development in mice. Surprisingly, deletion of *beclin*1 in *in vitro* human cells did not block an autophagy response, but attenuated the expression of several DNA double-strand break (DSB) repair proteins and formation of repair complexes, and reduced an ability to repair DNA in the cells exposed to ionizing radiation (IR). Overexpressing Beclin 1 improved the repair of IR-induced DSB, but did not restore an autophagy response in cells lacking autophagy gene *Atg7*, suggesting that Beclin 1 may regulate DSB repair independent of autophagy in the cells exposed to IR. Indeed, we found that Beclin 1 could directly interact with DNA topoisomerase IIβ and was recruited to the DSB sites by the interaction. These findings reveal a novel function of Beclin 1 in regulation of DNA damage repair independent of its role in autophagy particularly when the cells are under radiation insult.

Beclin 1 was initially identified as a Bcl-2-interacting coiled-coil protein that was expected to play a role in antiviral host defenses^1^. Soon after, the same group proposed Beclin 1 as the first mammalian autophagy protein^2^. Since its discovery, Beclin 1 has been the most extensively investigated mammalian autophagy protein. It acts as a core component of the Class III Phosphatidylinositol 3-Kinase Vps34 complex, which is essential to autophagosome formation and the initiation of autophagy^3^,^4^. Studies have also shown that Beclin 1 interacts with an increasing number of cofactors, including Atg14L, UVRAG, Bif-1, Rubicon, Ambra1, HMGB1, nPIST, VMP1, SLAM, IP(3)R, PINK and survivin, to regulate the lipid kinase Vps34 and to promote the formation of Beclin 1-Vps34-Vps15 core complexes, which induces autophagy^5^,^6^,^7^,^8^,^9^,^10^,^11^. These findings establish that Beclin 1 plays a central role in mammalian autophagy.

Beclin 1 has also been identified as a haploinsufficient tumor suppressor in mice and the human *beclin*1 gene is frequently and monoallelically deleted or expressed at reduced levels in breast, ovarian and prostate cancers^1^,^12^,^13^,^14^,^15^. We found that *beclin*1 is mutated in human leukemia cells^16^ and expressed at reduced levels in human CD34^+^CD38^−^ hematopoietic stem cells in acute myeloid or acute lymphoblastic leukemia patients^17^. In mice, the monoallelic depletion of *beclin*1 caused chromosomal instability through increased DNA damage, which led to tumor progression^18^. Oncogenic Akt signaling regulates the Beclin 1 complex and thereby leads to the inhibition of autophagy and tumorigenesis^19^. Intriguingly, previous studies showed that Beclin 1 plays a role in mitosis^20^, and that Beclin 1 bridges autophagy, apoptosis and differentiation^21^. However, the autophagic and tumor suppressive roles of Beclin 1 remain a major research focus, and the exact mechanism by which Beclin 1 acts as a tumor suppressor has not yet been fully understood. Studies on Beclin 1 have so far overwhelmingly supported the notion that its anti-tumor role is mediated through its involvement in autophagy^19^,^22^,^23^,^24^,^25^. Nevertheless, it remains an open question whether the tumor suppressive phenotypes associated with *beclin*1 deletions are a solely direct consequence of defective autophagy or whether they possibly indicate the involvement of alternative, previously unidentified functions of Beclin 1.

Here, we describe a distinct role of Beclin 1 in DNA damage repair independent of autophagy. While nutritional stress triggers an increase in the cytoplasmic localization of the protein for execution of autophagy, Beclin 1 is also triggered during development and especially when a life is in jeopardy to relocalize to the nucleus. Nuclear Beclin 1 protects genomic integrity by collaborating with DNA topoisomerase IIβ during DNA damage repair.

## Results

### Beclin 1 is progressively relocalized to the nucleus during development

**Figure 1:**
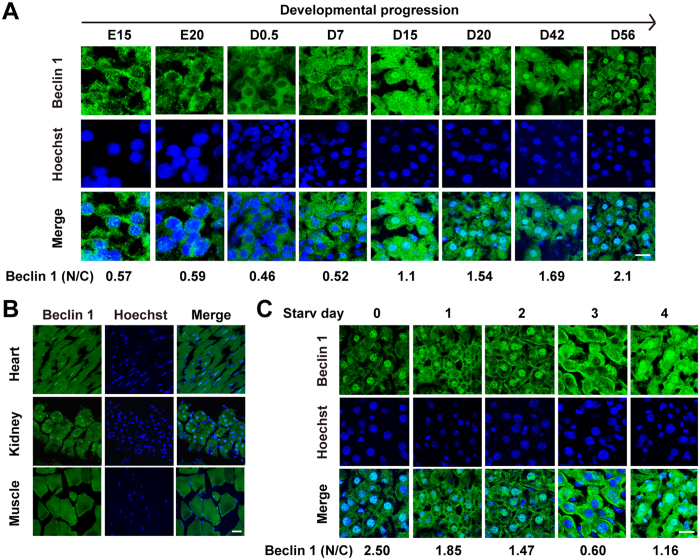
Beclin 1 is progressively relocalized to the nucleus during development and its nuclear distribution was reversed by starvation. (**A**) Representative microscopic images of the subcellular localization of Beclin 1 in hepatic cells at different stages of mouse development are shown. Beclin 1 was labeled using DyLight 488 antibodies, and nuclei were stained using Hoechst 33342. The ratio between nuclear Beclin 1 (N) and cytoplasmic Beclin1 (**C**) was quantified in ≥50 cells using TCS-SP2 software and presented at the bottom of the images. (**B**) Representative microscopic images of the subcellular localization of Beclin 1 in the heart, kidney, and muscle tissues from adult C57BL/6J mice (6–8 weeks old). (**C**) Representative microscopic images of Beclin 1 subcellular localization in adult mouse hepatocytes during 4 days of starvation (Starv). The ratio of nuclear Beclin 1 (N) and cytoplasmic Beclin1 (**C**) was presented at the bottom of the images. Similar results were observed in at least three independent experiments. Scale bar, 20 μM.

The *in vivo* subcellular distribution of Beclin 1 has not been described in the literature. To explore the localization of Beclin 1 in cells under physiological conditions, we examined the subcellular distribution of Beclin 1 in hepatic cells at different developmental stages. The results from our confocal microscopic analysis showed that during embryonic development and in neonatal mice, Beclin 1 (labeled green) is primarily located in the cytoplasm and plasma membrane, with a very small portion located in the nucleus (labeled blue) (Fig. 1A). There after, Beclin 1 progressively redistributed into the nucleus. When the mice were 15 days old, roughly half of the total Beclin 1 in hepatocytes was located in the nucleus. At postnatal day 20, the majority of Beclin 1 relocated into the nucleus, and much less Beclin1 remained in the cytoplasm. This finding was confirmed by immunoblot, which demonstrates that while total cellular levels of Beclin 1 were relatively stable during the course of mouse development (Fig. S1A), Beclin 1 relocated from cytoplasma to nucleus within a few weeks after birth (Fig. S1B). This pattern, in which an increased amount of Beclin 1 was localized in the nucleus, was similarly sustained in adult mice not only in hepatocytes but also in other tissues such as the heart and kidney (Fig. 1B).

We hypothesize that the lack of nuclear relocalization of Beclin 1 during neonatal period may be attributed a sudden interruption in the trans-placental supply of nutrients, which causes increases in autophagy for adaptation of the disruption in the maternal supply of nutrients^26^. To test this hypothesis, we starved adult mice for up to four days and then examined the distribution of Beclin 1 in mouse hepatic cells by confocal microscopy. The data show that as starvation progressed, nuclear Beclin 1 gradually decreased, while cytoplasmic Beclin 1 accordingly increased, until a small portion of the total cellular Beclin 1 remained in the nucleus. Surprisingly, on day after extreme starvation was induced, when the mice were about to die, the cytoplasmic relocalization of Beclin 1 rapidly reversed, and the overwhelming majority of cellular Beclin 1 re-translocated into the nucleus (Fig. 1C). This result was confirmed by Western blot analysis (Fig. S1C). These findings prompted us to explore how nuclear localization of Beclin 1 is regulated at the molecular level and whether Beclin 1 plays a more pivotal role in the nucleus than in the cytoplasm.

### Domains including residues 1–50 and 254–278 are involved in Beclin 1 nuclear localization

Beclin 1 contains three distinct functional domains, including an N-terminal Bcl-2 homology 3 (BH3)-only domain, a central coiled-coil domain (CCD) and a carboxy-terminal evolutionarily conserved domain ECD)^27^. No nuclear localization sequence was found in Beclin 1 1,28, nor did our search using computer software suggest the presence of a putative nuclear localization sequence in Beclin1. To understand how Beclin 1 is localized to the nucleus, we performed domain mapping of Beclin 1 by constructing a series of *beclin*1 mutants in which *beclin*1 was fused with a HA-tag to determine which region is potentially responsible for its nuclear localization (Fig. S2A). NIH3T3 mouse embryo fibroblast cells were transfected with these *beclin*1 mutants, and stable cell lines were generated using G418 selection. Western blot analyses of respective cytoplasmic and nuclear proteins suggest that protein domains including residues 1–50 and 254–278 are involved in Beclin 1 nuclear localization, and presence of either of the domains was sufficient for nuclear localization (Fig. S2B). The separation of cytoplasmic and nucleic proteins is successful (Fig. S3E). Mutation of the phosphorylation sites in Beclin 1, either individually or in combination, failed to hinder its nuclear localization (data not shown).

### Loss of Beclin 1 resulted in reduced DNA damage repair

Since constitutive deletion of *beclin*1 gene is embryonically lethal^14^, we generated Becn1^f/f^;Ubc-Cre mice to set out to investigate the role of Beclin 1 in the nucleus. Surprisingly, inducible deletion of *beclin*1 gene in young mice by tamoxifen caused immediate mouse death (data not shown). We thus shifted to construct a *beclin*1 knockout HeLa cell line using a CRISPR/Cas9 system to study the role of nuclear Beclin 1. Cas9 plasmids containing a guide RNA (gRNA) that targeted exon 5 and exon 6 of *beclin*1 (Fig. S3A) were transfected into Hela cells, and single cell clones were analyzed. A total of 18 randomly selected clones that harbored a gRNA targeting *beclin*1 were analyzed using polymerase chain reaction to show mutant bands in gel electrophoresis (Fig. S3B). Beclin 1 expression was undetectable in 5 clones that were examined using Western blot analysis (Fig. S3C). Sequencing analysis of these 5 clones demonstrated that the loss of Beclin 1 expression resulted from multiple indels that occurred at the targeted locus in *beclin*1 (Fig. S3D), which led to frame shifts and a loss of gene function.

**Figure 2:**
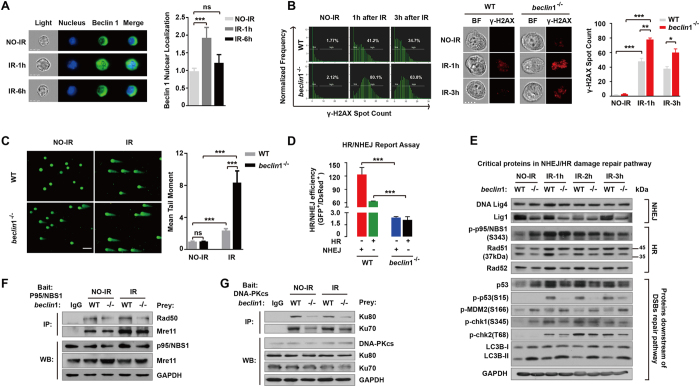
Loss of Beclin 1 resulted in reduced DNA damage repair. (**A**) Nuclear localization of Beclin 1 was analysed using image flow cytometry with or without 6 Gy irradiation. The nuclear localization index was determined using the manufacturer’s software (left), and representative images are shown (right). (**B**) γ-H2AX spot counts in both wild-type and beclin1-deleted Hela cells analyzed using image flow cytometry in cells with or without 6 Gy irradiation. The quantification of γ-H2AX spot counts was determined using the manufacturer’s software (left), and representative images are shown (middle). (**C**) A neutral Comet assay performed in wild-type and beclin1-deleted Hela cells with or without 6 Gy irradiation. Scale bar, 5 μM (left). Tail moments were calculated using Comet assay software in ≥50 cells (right). (**D**) Plasmid-based assays measuring HR and NHEJ activity in wild-type and beclin1-null cells. (**E**) Critical proteins involved in the NHEJ and HR DNA DSB repair pathways and critical proteins known to be downstream of the damage repair pathways were analyzed using immunoblotting. (**F**) Co-immunoprecipitation and immunoblotting analysis detecting MNR complex (including Mre11, p95/NBS1 and Rad50) in wild-type and beclin1 knockout cells treated with or without IR. p95/NBS1 was used as the bait protein. (**G**) Co-immunoprecipitation and immunoblotting analysis detecting DNA-PK complex (including DNA-PKcs, Ku70 and Ku80) in wild-type and beclin1 knockout cells treated with or without IR. DNA-PKcs was used as the bait protein. These experiments were repeated at least three times. The data presented in the bar graphs are mean values ± SEM from 3 or more independent experiments (right). *p < 0.05; **p < 0.01; *** and p < 0.001. (Full-length gels are included in the Supplementary Information file).

Exposure to ionizing radiation (IR) caused a transient increase in nuclear localization of Beclin 1 (Fig. 2A), suggesting that Beclin 1 may be involved in regulation of DNA damage response to radiation. In consistent with this hypothesis, we found that the *beclin*1 knockout cells showed a marked increase in the cells with the Ser139-phosphorylated histone H2AX (γ-H2AX) foci that are considered as a biomarker of DNA DSBs at 1 h post IR, compared with wild-type cells. More importantly, the spot counts of γ-H2AX foci by imaging flow cytometry at 1 h, 3 h, and 6 h after exposure to IR demonstrated that the *beclin*1 knockout cells also had a slower kinetics in the repair of IR-induced DNA DSBs than wild-type cells (Fig. 2B), indicating that the *beclin*1 knockout cells are deficient in the repair of DNA DSBs. This suggestion was further confirmed by the neutral comet assays at single cell levels (Fig. 2C). Mitigation of DNA injury via Beclin 1 may be applied to certain types of triggers since deletion of *beclin*1 in HeLa cells also increased sensitivity to DNA damage triggered by etoposide but not UV irradiation (Fig. S4).

DNA DSBs can be repaired by the HR and NHEJ pathways. To determine which of these pathways might be regulated by *beclin*1, we performed the HR and NHEJ repair reporter assays^29^,^30^. We found that knocking out of *beclin*1 led to a significant reduction in the repair of DNA DSBs by both of these pathways (Fig. 2D). Specifically, the *beclin*1 knockout cells showed approximately 60-fold and 30-fold reductions in DNA DSB repair by the NHEJ and HR pathways, respectively, compared with wild-type cells (Fig. 2D). This observation was further confirmed in a Western blot assay to measure the levels of critical proteins in these DSB damage repair pathway. The results showed that the *beclin*1 knockout cells expressed a lower level of the NHEJ repair protein DNA Ligase 1 than wild-type cells. Similarly, the *beclin*1 knockout cells exhibited lower levels of the HR repair proteins Rad 51 and Rad 52, and phosphorylated p95/NBS1 in response to IR, than wild-type cells (Fig. 2E). Furthermore, while the downstream proteins in the DNA DSB repair pathways, including p53, Chk1 and Chk2, were phosphorylated in wild-type cells in response to IR, these phosphorylation events were significantly reduced in the *beclin*1 knockout cells. In contrast, the *beclin*1 knockout cells expressed a higher level of the phosphorylated MDM2, which negatively regulates DNA damage response via induction of p53 degradation, than wild-type cells after IR (Fig. 2E). Notably, both the protein levels of LC3-I and LC3-II, and the LC3-II/LC3-I ratio increased in *beclin*1 knockout cells regardless of irradiation, suggesting either an increase in conversion of LC3-II from LC3-I or a decrease in degradation of LC3-II (Fig. 2E). This raises questions as to whether functional autophagy remains in the *beclin*1 knockout cells and whether changes in DNA damage repair proteins link to possible alteration in autophagy activity.

The MNR complex consisting of Mre11, p95/NBS1 and Rad50 and DNA-PK complex consisting of DNA-dependent protein kinase and Ku family of proteins play pivotal roles in the initiating the process of DSB repair by HR and NHEJ, respectively^31^,^32^. We performed co-immunoprecipitation and immunoblotting analysis to determine whether Beclin 1 regulates the formation of the complexes. The results from this analysis showed that wild-type cells are able to form both the MNR complex and DNA-PK complex, but knockout of *beclin*1 significantly attenuated the formation of the DNA-PK complex, with slight effect on the MNR complex (Fig. 2F,G), suggesting that Beclin 1 may regulate DNA DSB repair in part by promoting the formation of the DNA-PK complex and MNR complex.

As expected, reintroducing Beclin 1 into the *beclin*1-null cells significantly restored the cells’ capacity in radiation-induced DNA damage repair (Fig. S5), but *beclin*1 mutant lacking nuclear localization domains (residues 1–50 and 254–278) was unable to restore the wild-type cell phenotype in DNA damage repair (Fig. S6). These results further support a robust role of Beclin 1 in DNA damage repair, which depends on its nuclear localization.

### Beclin 1 regulates DNA DSB repair independent of autophagy

**Figure 3:**
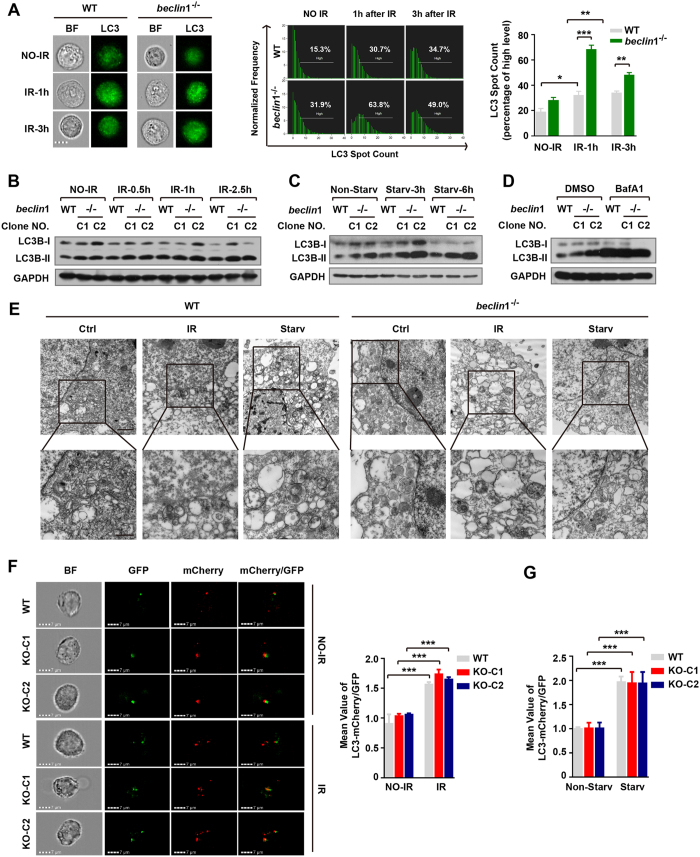
Loss of Beclin 1 did not impair functional autophagy. (**A**) Image flow cytometric analysis of autophagy activity. LC3 was labeled using FITC-conjugated anti-LC3 antibodies, and the green LC3 puncta were measured using Amnis ImageStream X Mark II Amnis IDEAS 4.0 software. Original magnification, ×600 (left), representative data (middle) and quantitative data (right) are shown. Scale bar, 7 μM. (**B**) Immunoblotting analysis of LC3b-I and LC3b-II in the indicated cells that were irradiated (IR 6 Gy). (**C,D**) Immunoblotting analysis of the conversion of LC3-I to LC3-II in wild-type and beclin1-null Hela cells treated with or without starvation (DMEM without serum) or bafilomycin A1 (Baf-A1, 25 nM). (**E**) Electron micrographs showing the ultrastructure of the control cells and the cells serum-starveted or irradiated. Both wild-type and beclin1-deleted Hela cells are shown. Scale bars in the upper panel, 2 μm; in the lower panel, 500 nm. (**F,G**) Autophagic flux analysis with LC3-mCherry-GFP plasmid. The plasmid was transfected into wild-type and *beclin*1-null HeLa cells. Stable cell lines expressing LC3-mCherry-GFP were sorted by BD FACS cytometry. Images of mCherry and GFP spots in both wild-type and *beclin*1-null HeLa cells were taken by image flow cytometry with 6 Gy irradiation or starvation for 3 hours in HBSS (lower). Representative images of irradiation were shown (**F**, left), and quantitative data were shown in **F** (right) and **G**. These experiments were repeated at least three times. The data presented in the bar graphs are mean values ± SEM from at least three independent experiments. *p < 0.05; **p < 0.01; ***p < 0.001. (Full-length gels are included in the Supplementary Information file)

To determine whether Beclin 1 regulates DNA DSB repair in an autophagy-dependent manner, we examined whether *beclin*1 deletion affects the autophagy response using image flow cytometry to visually and statistically analyze LC3b spot counts, a marker used for measuring the formation of autophagasomes. The results showed that there was a significantly higher number of LC3b puncta in the *beclin*1^−/−^ cells than in the wild-type cells at both the basal level and after IR exposure (Fig. 3A). To further evaluate the autophagy response after exposure to IR, we randomly chose two *beclin*1 knockout clones (KO15 and KO40) and exposed the cells to 6 Gy of IR. We then examined the dynamics of LC3b by analyzing the levels of lipidated LC3b (LC3b-II), a marker of autophagosomes that is converted from its unconjugated form (LC3b-I). The results showed that lacking *beclin*1 did not substantially change LC3b lipidation in cells exposed to IR, as manifested by the ratio between LC3b-II and LC3b-I after the data were normalized to the internal loading control (Fig. 3B). Surprisingly, when cells were serum-starved or exposed to bafilomycin A1, the LC3b-II/LC3b-I ratio in the *beclin*1 knockout clones was similar to the ratio in wild-type HeLa cells (Fig. 3C,D). These results suggested that autophagic flux was possibly maintained in the *beclin*1 knockout cells. We next examined the structure of autophagosomes using electron microscopy. The results showed that autophagic structures including double-membrane autophagosomes were observed in both the *beclin*1-deleted and the wild-type cells when cells were exposed to starvation or exposed to IR (Fig. 3E). To further support the above observations, we transfected LC3-mCherry-GFP plasmid into wild-type and *beclin*1-null HeLa cells, and sorted the cells for stable transfectants by BD FACS cytometry. After the sorted cells were starved for 3 hours, or irradiated for 5 Gy, the ratio of mean value between mCherry and GFP, which indicates a level of fusion between autophagosomes and lysosomes and thus reflects autophagic flux, was detected by flow cytometry. Since the mCherry/EGFP ratio was also increased upon starvation or irradiation in *beclin*1-deleted HeLa cells, comparable to wild-type cells (Fig. 3F,G), the results indicate that autophagic flux was maintained in *beclin*1-deleted cells. Our data thus suggest that functional autophagy remain intact when *beclin*1 was deleted while DNA DSB repair is dependent on Beclin 1. These results are in contrast to the results of a recent study that reported that deletion of *beclin*1 using TALEN disrupted autophagic flux by disrupting the formation of autophagosomes but not by disrupting LC3b lipidation in human Hela cells^33^. However, the present data appear to be consistent with our recent finding that somatic cells, but not stem cells, can acquire an alternative form of autophagy when their canonical autophagy pathway is disrupted^34^,^35^.

To further examine whether Beclin 1 regulates DNA DSB repair in an autophagy-dependent manner, we examined the effect of Beclin 1 on DNA DSB repair in cells that lack ATG7-dependent autophagy using *Atg7*-knockout K562 leukemia cells. We found that deletion of *Atg7* led to an increase in the number of γ-H2AX foci in cells before and after exposure to IR, which was attenuated by overexpressing Beclin 1 (Fig. S7A). Western blot analyses showed that the loss of ATG7 led to failures in LC3 lipidation and accumulation of p62. However, overexpressing Beclin 1 failed to rescue the autophagy response in *Atg*7-deleted cells because LC3 remained unlipidated and p62 was not reduced after overexpression of Beclin 1 in the cells with or without exposure to irradiation that is a known trigger for activation of autophagy (Fig. S7B). These results provide additional support to our hypothesis that Beclin 1 regulates DNA DSB repair independent of autophagy.

Interestingly, deletion of *beclin*1 caused upregulation of ULK1 regardless of imposed stresses including radiation and starvation (Fig. S8A,B), and image flow cytometric analysis further showed that loss of Beclin 1 resulted in the formation of ULK1 foci and increased the colocalization between ULK1 and LC3, and the ULK1 foci and ULK1-LC3 colocalization were augmented in response to irradiation (Fig. S8C). These suggest that an alternative autophagy may be activated when *beclin*1 is deleted because ULK1 was upregulated and recruited to autophagosome assembly site during activation of an alternative autophagy^34^,^35^.

### Beclin 1 promotes DNA DSB repair via a direct interaction with DNA topoisomerase IIβ

**Figure 4:**
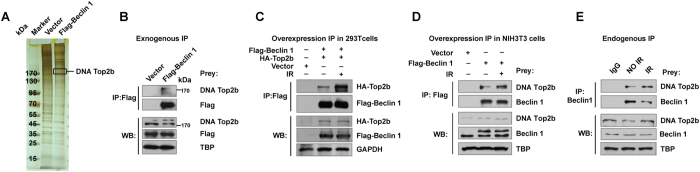
Beclin 1 binds to DNA topoisomerase IIβ in the nucleus. (**A**) Silver-stained SDS-PAGE revealed the Beclin1-interacting proteins were immuno-isolated from NIH3T3 cells that stably expressed Flag-tagged Beclin 1 or vector using anti-Flag beads. The proteins in the gel bands were extracted and identified using mass spectrometry, and DNA topoisomerase IIβ were detected. (**B**) Exogenous co-immunoprecipitation detecting DNA topoisomerase IIβ and Beclin 1 in nuclear lysates of NIH3T3 cells that stably expressed Flag-tagged Beclin1 or vector. The samples were subjected to immunoprecipitation analysis using anti-Flag antibodies. TBP was used as an internal control for nuclear samples. (**C**) Exogenous immunoprecipitation detecting DNA topoisomerase IIβ in 293T cells that overexpressed Flag-tagged Beclin 1 and treated with or without IR. (**D**) Exogenous immunoprecipitation detecting DNA topoisomerase IIβ in NIH3T3 cells that overexpressed Flag-tagged Beclin 1 treated with or without IR. (**E**) Endogenous co-immunoprecipitation and immunoblotting analysis detecting DNA topoisomerase IIβ and Beclin 1 in Hela cells treated with or without IR. All data are representative of the results of three independent experiments. (Full-length gels are included in the Supplementary Information file).

To elucidate the mechanism by which Beclin 1 exerts its effect on DNA DSB repair, we pulled down nuclear proteins associated with Beclin 1 in NIH3T3 cells which ectopically expressed Flag-tagged Beclin 1. The identities of the Beclin 1-interacting proteins were determined using mass spectrometry analysis (Fig. 4A), which identified DNA topoisomerase IIβ as one of the major Beclin 1-interacting proteins in the nucleus. This finding was confirmed by immunoprecipitation, which demonstrated that epitope-tagged Beclin 1 was able to specifically bind to DNA topoisomerase IIβ (Fig. 4B). Furthermore, we verified a physical interaction between epitope-tagged Beclin 1 and DNA topoisomerase IIβ in both human embryonic kidney 293T cells and murine fibroblasts NIH3T3 cells (Fig. 4C,D), and between endogenous Beclin 1 and DNA topoisomerase IIβ in HeLa cells. We found that this interaction was intensified after exposure to IR (Fig. 4E). These results suggest that Beclin 1 may regulate DNA DSB repair via its interaction with DNA topoisomerase IIβ.

**Figure 5:**
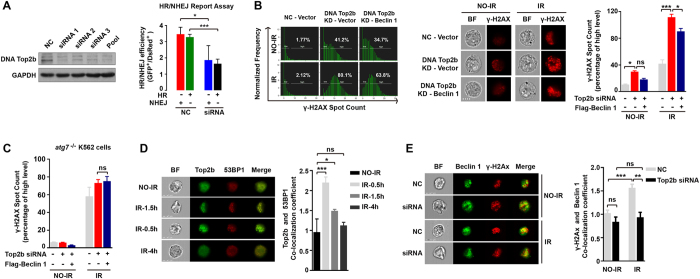
Depleting DNA topoisomerase IIβ attenuated the DNA damage repair capacity of Beclin 1. (**A**) Immunoblotting of DNA topoisomerase IIβ in Hela cells silenced with siRNAs targeting the gene encoding DNA topoisomerase IIβ (left). Plasmid-based assays measuring HR and NHEJ activity in wild-type and DNA topoisomerase IIβ siRNA-silenced Hela cells (right panel). (**B**) γ-H2AX spot counts analyzed using image flow cytometry after siRNA interference and transfection with Beclin 1 full-length plasmids (left). Representative flow images are shown. Scale bar, 7 μM (middle) and statistic results are shown (right). All bars show the mean values ± SEM as quantitated from ≥3 independent experiments. (**C**) γ-H2AX spot counts analyzed using image flow cytometry after siRNA interference of DNA topoisomerase IIβ and overexpression with Beclin 1 in Atg7-null K562 cells. (**D**) DNA topoisomerase IIβ and 53BP1 co-localization was analyzed using image flow cytometry at 0.5, 1.5, and 4 h after irradiation. The co-localization efficiency was determined using the manufacturer’s software (left) which is based on the similarity between two pixel, and representative flow images are shown (right). (**E**) Beclin 1 and γ-H2AX co-localization was analyzed using image flow cytometry after siRNA interference of DNA topoisomerase IIβ. Representative flow images are shown. Scale bar, 7 μM (left) and statistic results are shown (right). ns, not significant. *p < 0.05; **p < 0.01; ***p < 0.001. Data are representative of at least three independent experiments.

To test this hypothesis, we next explored whether DNA topoisomerase IIβ participates in DSB repair and whether Beclin 1 promotes DSB repair dependent on DNA topoisomerase IIβ. Knockdown of DNA topoisomerase IIβ significantly reduced the activities of the HR and NHEJ pathways (Fig. 5A). Furthermore, knockdown of DNA topoisomerase IIβ resulted in a higher percentage of irradiated cells with γ-H2AX foci than were observed in the cells with control siRNA. The increase in the number of radiation-induced DSBs was only partially mitigated by overexpressing Beclin 1 in the autophagy-intact DNA topoisomerase IIβ-depleted cells (Fig. 5B). To determine whether the effect of overexpressing Beclin 1 on DNA DSB repair is mediated by cytoplasmic Beclin 1 via the autophagy pathway or nuclear Beclin 1, we evaluated the effect of overexpressing Beclin 1 in *Atg*7^−/−^ autophagy defective cells in which DNA topoisomerase IIβ was silenced. In these experiments, overexpressing Beclin 1 did not restore the ability of *Atg*7^−/−^ autophagy defective cells to repair DNA DSBs when topoisomerase IIβ was depleted in the cells (Fig. 5C). These results confirm that the role of Beclin 1 in promoting DSB repair relies on the presence of DNA topoisomerase IIβ.

Furthermore, after exposure to IR, DNA topoisomerase IIβ and Beclin 1 were found to be colocalized with the DSB repair protein p53 binding protein 1 (53bP1)^36^ (Fig. 5D) or γ-H2AX (Fig. 5E). Strikingly, depletion of DNA topoisomerase IIβ by gene silencing completely prohibited Beclin 1 from localization to the DNA break sites (Fig. 5E), suggesting that DNA topoisomerase IIβ may recruit Beclin 1 to the DNA damage site through its direct interaction to Beclin 1 to promote DNA damage repair.

## Discussion

Recent studies by our team and other groups have suggested that ATG7-dependent autophagy may be linked to the DNA damage repair pathway because inducing the loss of autophagy by deleting the autophagy-essential gene *Atg*7 results in the downregulation of certain DSB repair proteins^37^,^38^. In these studies, the activation of autophagy by rapamycin elevated the levels of DNA repair proteins in both HR and NHEJ pathways, and loss of *Atg*7 resulted in a decrease in DSB repair activity^37^ and an increase in the proteasomal degradation of checkpoint kinase 1, which is a critical factor in HR repair pathway^38^. Autophagy is, in essence, a metabolic pathway that involves lysosomal degradation^39^. Although recent studies have reported that chaperone-mediated autophagy protects genomic stability by regulating the degradation of activated checkpoint 1 protein following genotoxic insult^40^ and although autophagy promotes nucleotide excision repair by enhancing DNA damage recognition^41^, the direct targets of autophagic degradation, in particular macroautophagic degradation that might connect these DNA repair cascades have not yet been identified. This raises the question of whether DNA repair is a direct consequence of the activation of the macroautophagic degradation program or whether it reflects a concurrent event in which the activation of macroautophagy by an autophagy inducer activates certain autophagy proteins that in turn regulate DNA damage repair through a potentially autophagy-independent process. Beclin 1 was found to improve DNA stability by physical interaction to UVRAG in human colorectal cancer cells in transient knockdown experiments^42^, but whether this effect is mediated by autophagy pathway was not identified, albeit a previous report showing autophagy-independent role of UVRAG in maintaining DNA stability^43^.

Our work in this study establishes that Beclin 1 plays an autophagy-independent role in maintaining genomic integrity by promoting a cell’s capacity for DNA damage repair. The findings challenge the long-standing paradigm that underlies our understanding of the mechanisms that are involved in the autophagy-mediated tumor suppressive role of Beclin 1. This study also reveals that Beclin 1 progressively relocalizes to the nucleus during mammalian development, and its nuclear localization is particularly increased when under either extreme starvation conditions or when exposed to nuclear radiation. We therefore propose that Beclin 1 plays a critical role in protecting the nucleus during development and alleviating extreme life-threatening levels of stress. The nuclear localization of Beclin 1 depends on two short domains covering residues 1～50 and 254～278. Loss of either domain disables Beclin 1 nuclear localization. Although we did not find indispensible phosphorylation site for Beclin 1 nuclear localization, whether other types of posttranslational modifications are involved in Beclin1 nuclear localization and whether upstream signals directly link to these essential domains for Beclin 1 nuclear localization remain to be resolved in the future.

**Figure 6:**
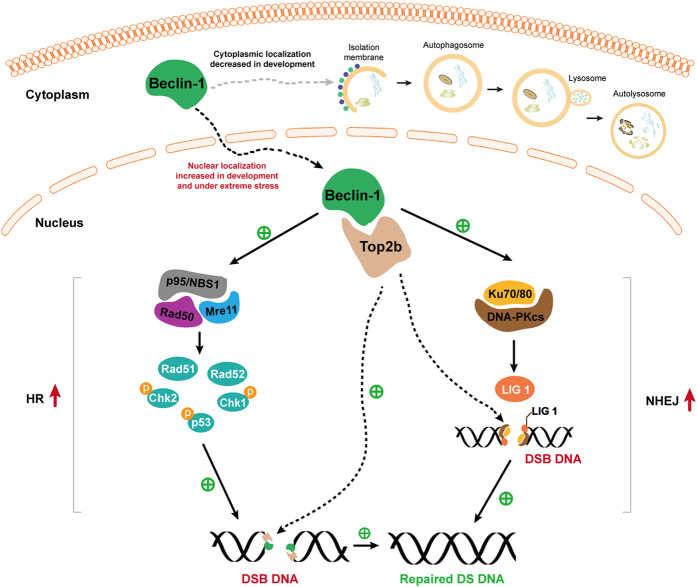
The diagram illustrating the role of Beclin 1 in DNA damage repair. Beclin1 progressively relocalizes to the nucleus during mammalian development, and its nuclear localization is particularly increased when under extreme stress. Beclin 1 plays a critical role in protecting the nucleus during development and alleviating extreme life-threatening levels of stress by promoting DNA DSB repair. The role of Beclin 1 in mitigating DNA damage depends on its interaction to DNA topoisomerase IIβ that recruits Beclin 1 to DNA damage site. Beclin 1 is indispensible for both the activation of DNA repair proteins and the formation of NR and DNA-PK repair protein complexes. The role of Beclin 1 in DNA damage repair is independent on autophagy-mediated pathway.

Furthermore, we show that Beclin 1 is implicated in both the HR and NHEJ DSB repair pathways because deleting *beclin*1 reduced their repair activity by 30- to 60-fold in a reporter assay. Deleting *beclin*1 also disabled the phosphorylation of several DNA repair proteins and the formation of MNR and DNA-PK complexes known to be critically involved in the DSB repair pathway. Finally, we show that DNA topoisomerase IIβ interacts with and recruits Beclin 1 to DNA break site in the nucleus during responses to nuclear radiation. While overexpressing Beclin 1 improved DNA repair in cells lacking ATG7-dependent autophagy, Beclin 1 failed to do so in cells when DNA topoisomerase IIβ was depleted. The partial rescue of the loss of topoisomerase IIβ in wild-type cells but not in autophagy-defective cells was contributed by enhanced autophagy due to overexpression of Beclin 1. In wild-type cells where autophagy is intact, overexpression of Beclin 1 enhances autophagy, which in turn improves DNA repair. In contrast, overexpression of Beclin 1 in autophagy defective cells no longer restores autophagy capacity, and mitigation of DNA damage by Beclin 1 solely depends on its interaction to topoisomerase IIβ. Therefore, when neither autophagy nor T topoisomerase IIβ is in place, Beclin1 is unable to improve DNA damage repair. A diagram summarizing our data is illustrated in Fig. 6. This finding suggests that when manipulating cytoplasmic Beclin 1 that is involved in the autophagy pathway in order to explore targeted therapies, the non-autophagy cytoprotective role of Beclin 1 in the nucleus should also be considered. Taken our recent finding that autophagy is radioprotetive^37^ and the present work on Beclin1 in DNA damage repair independence of autophagy together, combinatory activation of autophagy and upregulation of Beclin 1 may be contributable to a more effective mitigation of radiation injury.

## Methods

### Cell lines and cell culture

Cell lines HeLa, NIH3T3 and K562 were obtained from the ATCC and resuscitated from early passage liquid nitrogen stocks as needed. Cells were cultured for less than 3 months before reinitiating cultures and were routinely inspected microscopically for stable phenotype. Hela and NIH3T3 cells were cultured in DMEM, K562 cells in RIPM 1640, all supplemented with 10% fetal bovine serum at 37 °C, in a 5% CO2 incubator. For starvation experiments, cells were washed twice with PBS and then incubated for indicated time in Hanks balanced salt solution or DMEM without serum. For autophagic flux experiment, cells were grown at a density in log phase with bafilomycin A1 (Sigma–Aldrich) treatment for indicated concentration.

### Reagents and antibodies

Antibodies against Beclin1, Rad52, Histone H3, TBP, p53, p-p53(S15), p-Chk1(S345), p-Chk2(T68), p-MDM2(S166), PI3KC3, Bcl-2 for Western blotting analysis or co-immunoprecipitation assay were from Cell Signaling Technology. Beclin 1 antibody (Dylight 488) for fluorescent confocal analysis was from Novus Biologicals. Phospho-Histone H2AX (Ser139) monoclonal antibody (Alexa Fluor^®^ 647 Conjugate) for cytometric analysis was from Cell Signaling Technology. Antibodies targeting DNA Lig4, XLF, XCCR4, p-p95/NBS1(S343), Rad52, Rad51 for Western blotting analysis or co-immunoprecipitation assay were from Abcam. Anti-LC3b monoclonal antibody for Western blotting was from Novus Biologicals. Anti-Flag affinity isolated antibody was ordered from Sigma–Aldrich. GAPDH, DNA Top2b, DNA Lig1 polyclonal antibody were ordered from Proteintech. The horseradish peroxidase (HRP)-conjugated secondary antibodies were purchased from Cell Signaling Technology.

### Animals

C57BL/6J mice were sacrificed at the indicated time. For starvation experiments, mice were feed only with water. Male and female mice were used equally in all experiments and littermates were always used as controls. Each group contains at least 6 mice. All experiments with animals are complied with the institutional protocols on animal welfares and approved by the Ethics Committee of Soochow University.

### Fluorescence microscopy

Tissues isolated from C57BL/6 mice were excised and fixed in 4% paraformaldehyde at 4 °C for 16 h, and then soaked in 20% sucrose in PBS at 4 °C overnight. Specimens were frozen in JUNG tissue freezing medium(Leica), and 5 μm cryostat sections were cut, mounted onto glass slides coated with 0.01% Poly-L-Lysine (Sigma) and tissues were blocked with 5% goat serum in PBS for 60 min. NIH3T3 cells were grown on glass circular slips in 24-well plates. After treatment, cells were washed with PBS twice, fixed with pre-cooled 4% paraformaldehyde for 10 min at room temperature, permeabilized for 30 min with 0.25% Triton X-100 (Sigma) and blocked for 30 min in block buffer (1% FBS + 0.2% sapomin in PBS). All samples were incubated with the primary antibodies overnight at 4 °C and followed by the secondary antibody for 1 h at room temperature. The nuclear was stained with 20 μg/ml Hoechst 33342(Invitrogen) at room temperature for 10 min. The images were obtained by FV1200 (Olympus, Japan) confocal microscope system.

### ImageStream analysis

HeLa cells were treated with 6 Gy 60Co γ-ray irradiation with a dose rate of 1 Gy/min before visualized and analyzed with ImageStreamX Mark II (Amnis, Seattle, USA). Cells were gated with the area and aspect ratio features for single cells and gradient RMS features for focused cells. The LC3 and γ-H2AX spot counts were analyzed by spot count wizard with IDEAS 5.0 software (Amnis, Seattle, USA). Autophagy level and γ-H2AX foci were calculated by measuring cell percentage with high spot counts.

### Co-immunoprecipitation

Cells were lysed with IP lysis buffer (Beyotime Biotechnology) for total protein, and with NE-PER Nuclear and Cytoplasmic Extraction Kit (Pierce, Thermo Scientific) for cytoplasmic or nuclear protein. Then proteins were subjected for Co-IP according to the manufacturer’s protocol of Co-IP Kit (Pierce, Thermo Scientific), or incubated with 1–2 μg of the indicated antibodies at 4 °C for 8–12 h. After adding protein A/G (Pierce, Thermo Scientific), incubation was continued for an additional 2 h. For *in vitro* Flag and HA-tag pulldown, the whole-cell extracts were incubated with 50% anti-Flag M2 affinity gel (Sigma, A2220) overnight at 4 °C. The precipitates were then extensively washed with lysis buffer and eluted by boiling with SDS-PAGE loading buffer (eBioscience) for 5 min at 95 °C.

### Transfection and RNA interference

Full-length cDNA corresponding to the coding sequence of mouse or human *beclin*1 was obtained by polymerase chain reaction (PCR) and subcloned into PlinBin vector encoding N-terminal 3xflag and HA epitope tag. Truncation mutant fragments of *beclin*1 were amplified from the template *beclin*1 by PCR and subcloned into the PlinBin vector. HeLa and NIH3T3 stable cell lines were established by transfection followed by selection using 800 μg/ml or 500 μg/ml of G418 sulfate (Invitrogen) for 10 days. Small interfering RNA (siRNA) for human DNA topoisomerase IIβ (siRNA1:5′-GCAGUUCAUGUGGGUGUAUTT-3′, siRNA2: 5′-GGACAACAAGTTTGACCATGC-3′, siRNA3: 5′-GCTCAGTATCAGAGAGAATAC-3′) and negative control siRNA were designed by Genepharma (Shanghai, China). Hela cells were transfected with the siRNA duplexes using Lipofectamine 2000TM (Invitrogen) according to the manufacturer’s instructions with a final concentration of 30 nM. Cells were harvested 72 h after transfection and the efficiency was evaluated by immunoblotting.

### NHEJ & HR report assay

The NHEJ/HR reporter plasmid was used to determine the *in vivo* levels of NHEJ or HR pathway activity. 0.1 μg of plasmid encoding DsRed to control for transfection efficiency, together with 1 μg of plasmid encoding I-SceI endonuclease to induce DSBs, were cotransfected into the cells using Lipofectamine 2000TM (Invitrogen). GFP+ and DsRed+ were quantified by BD FACS Aria III 48 h after transfection. Efficiency of NHEJ or HR pathway was calculated by dividing the percentage of GFP-positive cells to the percentage of DsRed-positive cells. For each treatment, a minimum of 20,000 cells were analyzed by FACS. Data analysis was done using Flowjo software.

### Autophagic flux assay with LC3-mCherry-GFP plasmid

The plasmid was transfected into wild-type and *beclin*1-null HeLa cells with lipodosome. 72 hours after transfection, GFP and mCherry double positive cells were sorted with BD FACS machine according to the protocol previously reported^44^. Stable cell lines were starved for 3 hours in HBSS or exposed to a dose of 6 Gy irradiation. Images were taken by image flow cytometry.

### Electron microscopy

The cells were fixed in 2.5% glutaraldehyde in 0.1 M phosphate buffer, pH 7.2, at 4 °C. After 2 hours of fixation in osmium tetroxide (3%), cells were then dehydrated in graded acetone, and embedded in Araldite (Fluka). Ultrathin sections were prepared using a diamond knife, collected on copper grids (G300 Cu), contrasted using both lead citrate and uranyl acetate, and then examined with a Zeiss transmission electron microscope (JEOL-1010).

### Construction of knockout cell lines using CRISPR/Cas9 system

The *beclin*1 or *Atg*7 sgRNA sequence was cloned into the Cas9 vector provided by YSY biotech Company (Nanjing, China). The two targeting sequences for *beclin*1 were: sgRNA1 (5′-CAGCTTCACTCTGATTGGGGAGG-3′) targeting exon 5, and sgRNA2 (5′-CCAGACAGATGTGGATCACCCAC-3′) targeting exon 6. The two targeting sequences for *Atg*7 were: sgRNA1 (5′-CGTGAGACACATCACATTTGTGG-3′), and sgRNA2 (5′-CCAGAAAATATTCCCCGGTGTGg-3′) targeting exon 12. Annealing both single-stranded oligonucleotides resulted in a double-stranded oligonucleotide with compatible ends for cloning into the Cas9 vector that was then ligated into the vector using T4 DNA ligase. The two sgRNA-Cas9 ligated vectors were simultaneously transfected into cells. Single-cell derived colonies were isolated and the identification and verification of gene knockout were based on both sequencing analysis of genome PCR fragments of targeting locus and western blotting analysis using antibodies specifically targeting *beclin*1 or *Atg*7.

### Comet assay

Neutral pH comet assay was performed as described previously^35^.

### Statistical analysis

The data are presented as mean values from three separate experiments ± s.d. Statistical analysis are performed using GraphPad Prism 5 Software. Error bars represent SEM and p values calculated with a two-tailed Mann–Whitney test unless stated otherwise. (ns, no significance, *P < 0.05, **P < 0.01, ***P < 0.001).

## Additional Information

**How to cite this article**: Xu, F. *et al*. Nuclear localization of Beclin 1 promotes radiation-induced DNA damage repair independent of autophagy. *Sci. Rep.* **7**, 45385; doi: 10.1038/srep45385 (2017).

**Publisher's note:** Springer Nature remains neutral with regard to jurisdictional claims in published maps and institutional affiliations.

## Supplementary Material

Supplementary Information
