# Author Correction: Nuclear localization of Beclin 1 promotes radiation-induced DNA damage repair independent of autophagy

**DOI:** 10.1038/s41598-019-41388-7

**Published:** 2019-04-03

**Authors:** Fei Xu, Yixuan Fang, Lili Yan, Lan Xu, Suping Zhang, Yan Cao, Li Xu, Xiaoying Zhang, Jialing Xie, Gaoyue Jiang, Chaorong Ge, Ni An, Daohong Zhou, Na Yuan, Jianrong Wang

**Affiliations:** 1 Hematology Center of Cyrus Tang Medical Institute, Collaborative Innovation Center of Hematology, Soochow University School of Medicine, Suzhou, 215123 China; 2 Jiangsu Institute of Hematology, Jiangsu Key Laboratory for Stem Cell Research, The First Affiliated Hospital, Soochow University School of Medicine, Suzhou, 215123 China; 3 Division of Radiation Health, Department of Pharmaceutical Sciences, University of Arkansas Medical Sciences, Little Rock, Arkansas 72205 USA

Correction to: *Scientific Reports* 10.1038/srep45385, published online 27 March 2017

This Article contains errors. In Figure 2C, the representative comet image for Beclin^−/−^ IR group is incorrect. The correct Figure 2C appears below as Figure 1.

**Figure 1:**
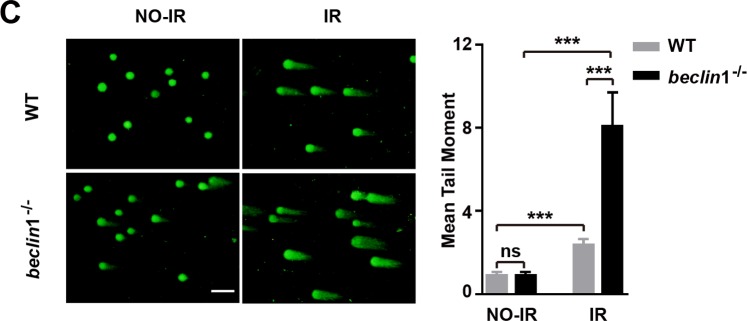
.

In Figure 5B, the image flow cytometry is incorrect. The correct Figure 5B appears below as Figure 2.

**Figure 2:**
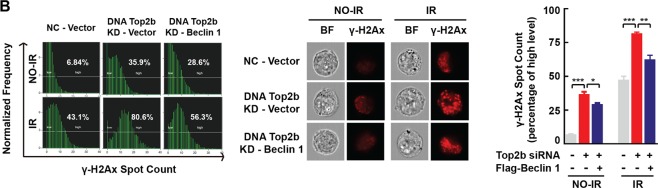
.

In the legend of Supplementary Figure S1,

“GAPDH served as an internal control for cytoplasmic protein loading in (C)”

should read:

“β-actin served as an internal control for cytoplasmic protein loading in (C)”

In the legend of Supplementary Figure S3,

“(**B**) Immunoblotting analysis of the indicated wild-type cells and the various beclin1 targeted Hela cell clones. GAPDH was used as an internal control in this and other figures”.

should read:

“(**C**) Immunoblotting analysis of the indicated wild-type cells and the various beclin1 targeted Hela cell clones. GAPDH was used as an internal control in this and other figures”.

In Supplementary Figure S5B, the representative image for beclin1^−/−^ Beclin1 IR-3h group is incorrect. The correct Supplementary Figure S5B appears below as Figure 3.

**Figure 3:**
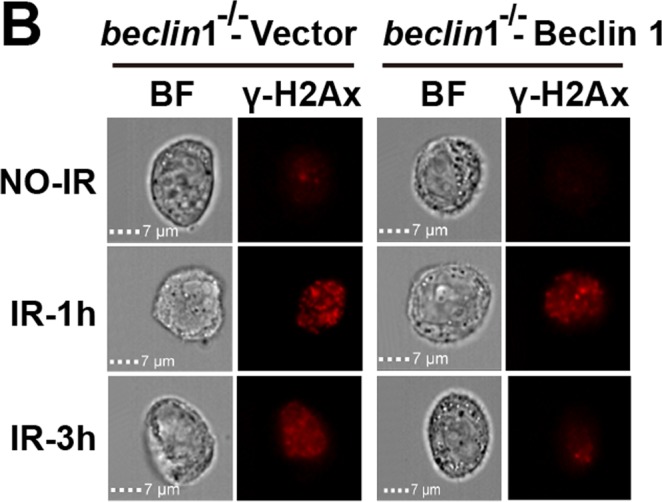
.

Finally, in Supplementary Figure S7A, the statistical comparison of the histogram between atg7^−/−^ Vector and atg7^−/−^ Beclin1 should be ‘**’, not ‘ns’”. The correct Supplementary Figure S7A appears below as Figure 4.

**Figure 4:**
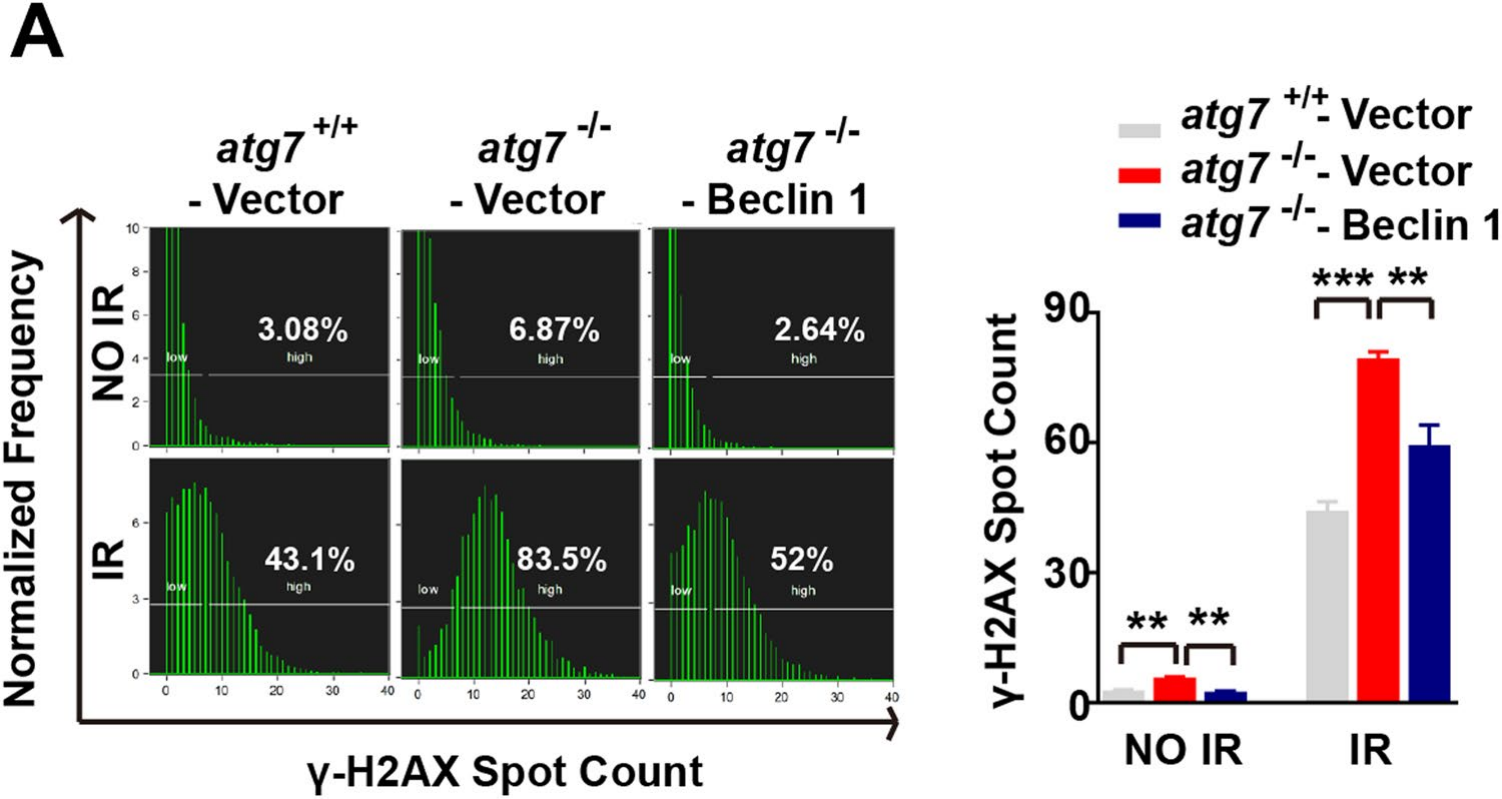
.

