# Supplementary material for: Nuclear localization of Beclin 1 promotes radiation-induced DNA damage repair independent of autophagy
Source: Sci Rep. 2017 Mar 27;7:45385. doi: 10.1038/srep45385 (PMC5366945; doi:10.1038/srep45385)
Supplement: Supplementary Information [file srep45385-s1.pdf]

Nuclear localization of Beclin 1 promotes radiation-induced DNA damage repair independent of autophagy

Fei Xu, Yixuan Fang, Lili Yan, Lan Xu, Suping Zhang, Yan Cao, Li Xu, Xiaoying Zhang, Jialing Xie, Gaoyue Jiang, Caorong Ge, Ni An, Daohong Zhou, Na Yuan, Jianrong Wang

Supplementary Information

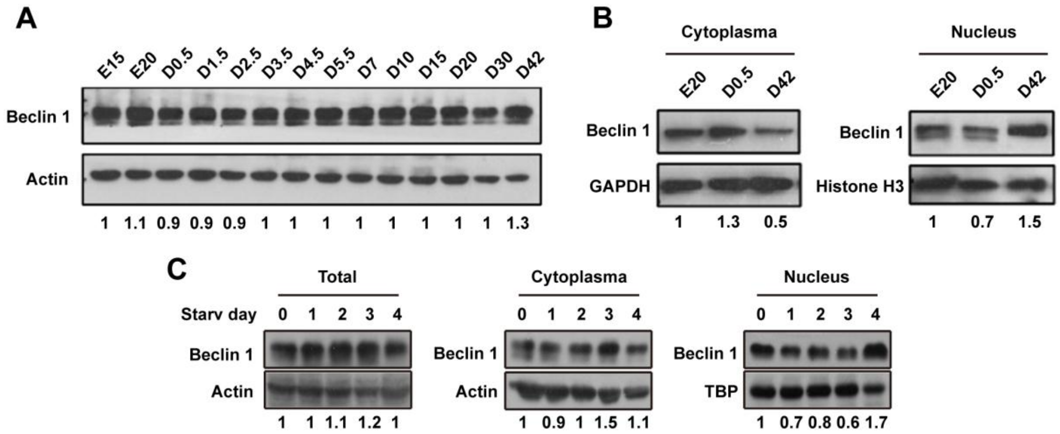

Figure S1. Subcellular distribution of Beclin 1 in mouse hepatocytes during development and after starvation. (**A**) A representative immunoblotting analysis of total Beclin 1 levels during mouse development from embryonic (E) day 15 to day (D) 42 after birth. (**B**) A representative immunoblotting analysis of cytoplasmic and nuclear Beclin 1 during mouse development. (**C**) Western blot analysis of total, cytoplasmic and nuclear Beclin 1 in hepatocytes from adult C57BL/6J mice on different days of starvation. β-actin served as an internal control for total protein loading in (A) and (C); GAPDH served as an internal control for cytoplasmic protein loading in (C); and Histone H3 and TBP served as an internal control for nuclear protein loading in (B) and (C), respectively. The data are representative of at least three independent experiments. The numbers presented in the bottom of each blot are the ratio of Beclin 1 vs. internal controls.

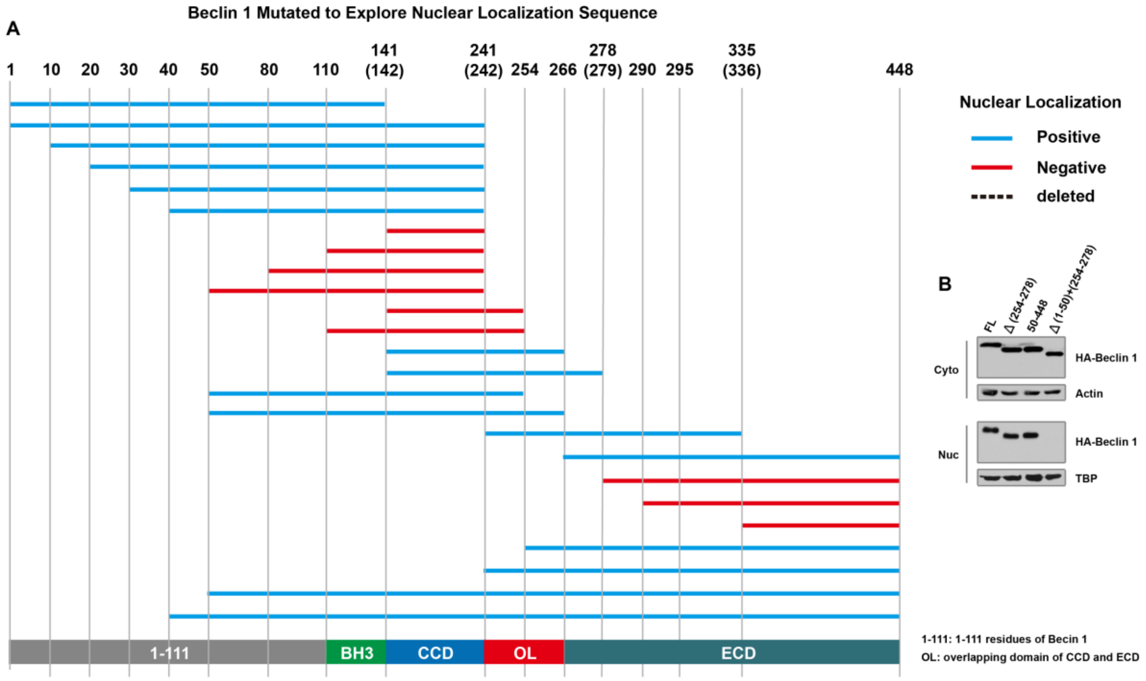

Figure S2. Both 1-50 and 254-278 residues are required for Beclin 1 localization to the nucleus. (**A**) Schematic diagram of the construction of the full-length Beclin 1 and Beclin 1 mutants in which different residues were deleted. The numbers above the schematic indicate amino acid positions. Mutants in blue represents positive nuclear localization, mutants in red represents negative nuclear localization, dotted lines represents truncate residues. Seen in the bottom is the organization of known functional domains of Beclin 1. (**B**) Representative Western blot showing impaired nuclear localization of Beclin 1 due to loss of domains covering residues 1-50 and 254-278.

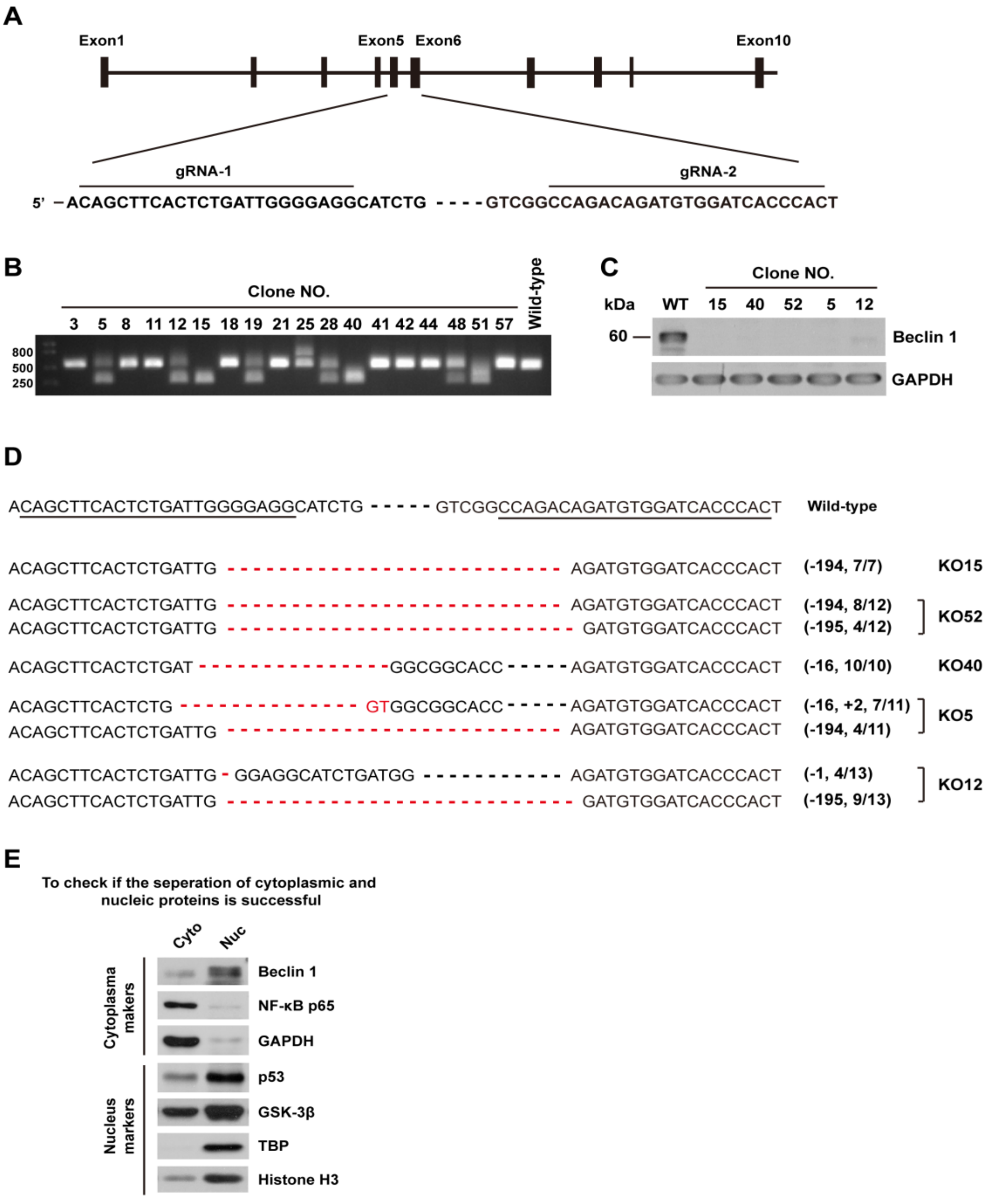

Figure S3. Deletion of the *beclin*1 gene. (**A**) The strategy used to knock out beclin1 with CRISPR/Cas9. Exon 5 and exon 6 were separately targeted using two siRNAs. The two siRNA-binding sequences are indicated by underlining at the appropriate locus. (**B**) PCR amplification was performed to detect the beclin1-containing targeted sequences, and the results were analyzed in agarose gels. (**B**) Immunoblotting analysis of the indicated wild-type cells and the various beclin1 targeted Hela cell clones. GAPDH was used as an internal control in this and other figures. (**D**) Sequence alignment to analyze the beclin1 gene in the genomes of the selected beclin1 knockout HeLa clones (#15, #52, #40, #5 and #12). The fraction number shown in the brackets indicate the number of genome PCR clones that were tested in each particular edited version. These were compared to the total number of PCR clones that were tested in the single cell-derived clones. (**E**) Immunoblotting detection to analyze several cytoplasmic and nucleic markers to determine whether the separation of cytoplasmic and nucleic proteins was successful.

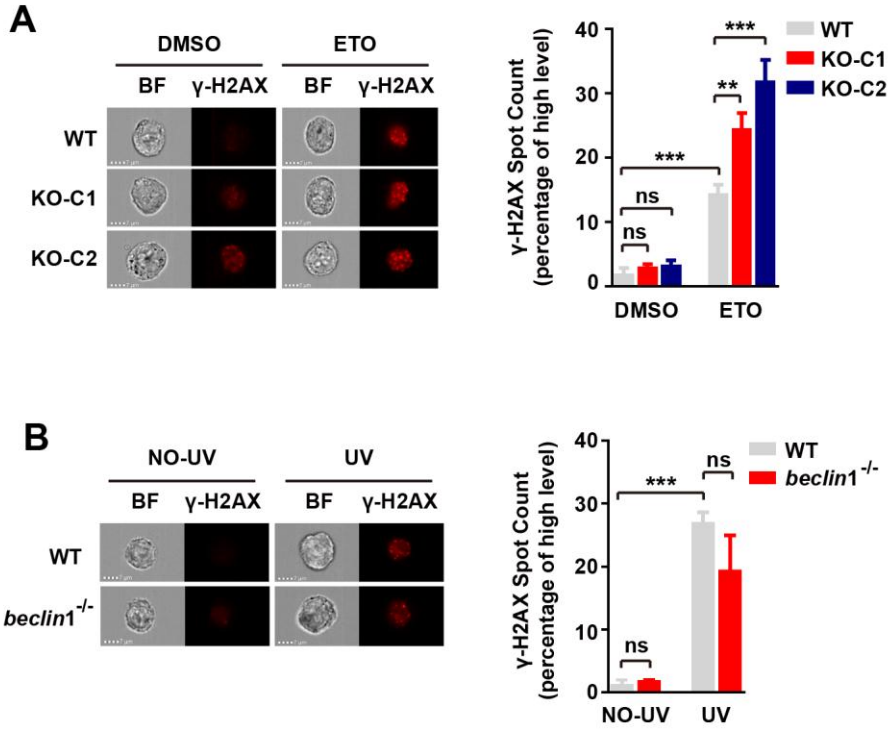

Figure S4. Beclin1-null cells show increased DNA DSBs when exposed to etoposide , but not UV irradiation. (**A**) Image flow analysis of wild-type and *beclin*1-deleted HeLa cells following treatment with etoposide (5ug/ml) for 2 hours. (**B**) Both wild-type and *beclin*1-deleted HeLa cells were UV irradiated (1mJ/cm^2) and were maintained for additional 30 min in cell culture incubator. γ-H2AX spot counts were evaluated by image flow cytometry. Representative flow images were shown, Scale bar, 7 μM. Quantification was determined by the manufacturer’s software. These experiments were repeated at least three times.

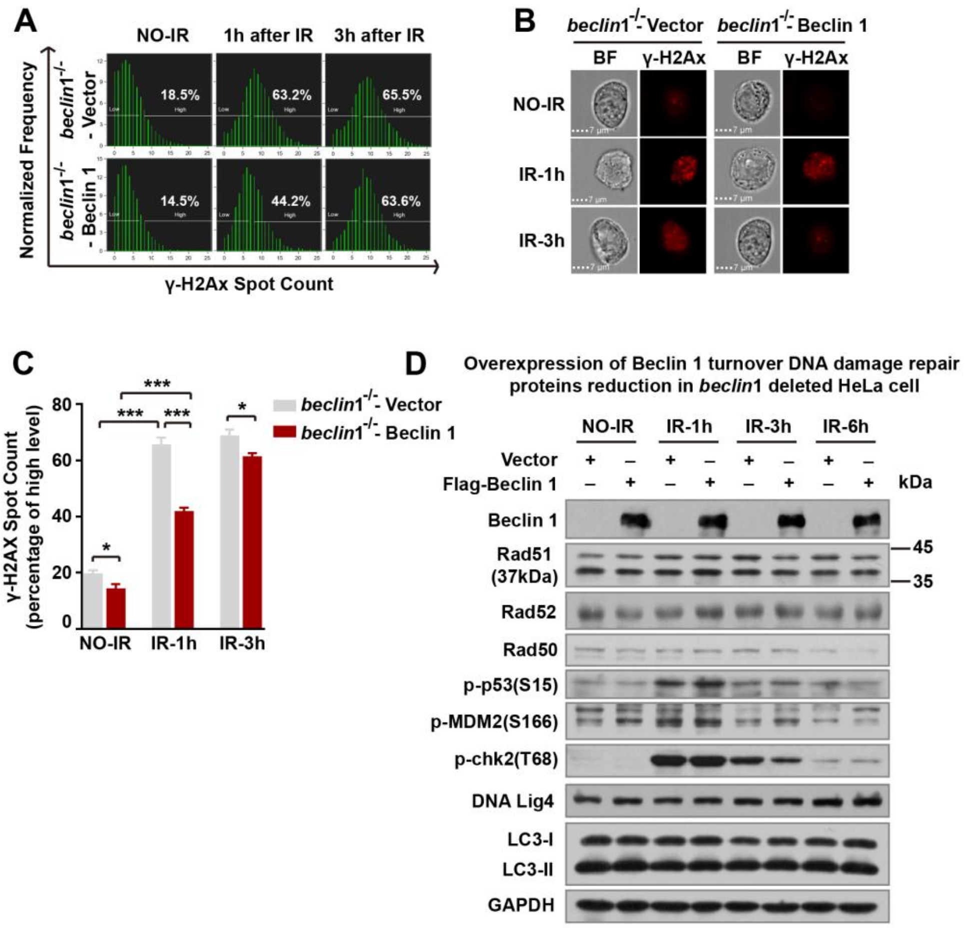

Figure S5. Overexpression of Beclin1 restores DNA damage repair capability in *beclin*1-deleted cells. (**A, B**) Vector and Beclin 1 full-length plasmid was transfected into *beclin*1-deleted HeLa cells. Images were taken by image flow cytometry of γ-H2AX spot counts after irradiation with indicated time. Representative flow images were shown. Scale bar, 7 μM. (**C**) Quantification was determined by the manufacturer's software. (**D**) Several important DSBs repair proteins were analyzed by immunoblotting with or without irradiation treatment. These experiments were repeated at least three times.

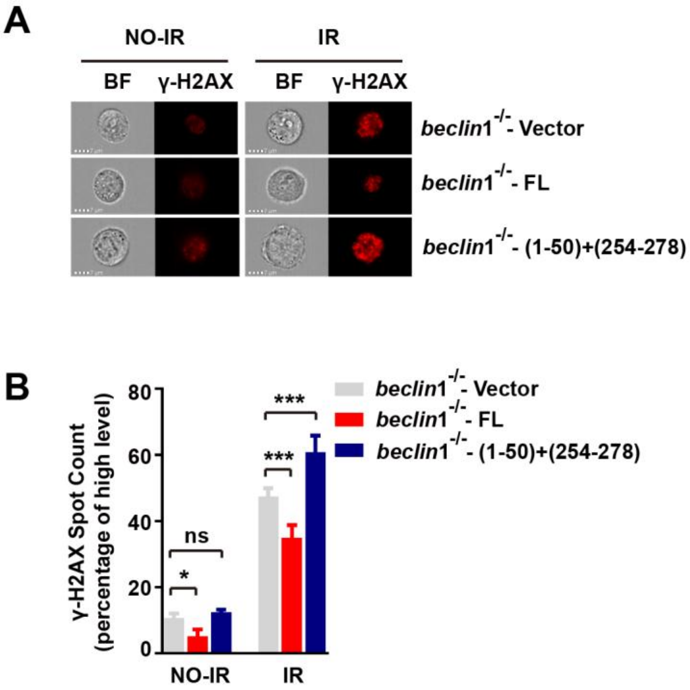

Figure S6. Expression of Beclin1 mutant lacking residues 1-50 and 254-278 failed to rescue DNA damage repair capacity in *beclin*1-deleted cells. (**A**) Beclin 1 full-length or Beclin1 mutant lacking residues 1-50 and 254-278 was transfected into *beclin*1-deleted HeLa cells. γ-H2AX spot counts were evaluated by image flow cytometry 3 hours after 6 Gy irradiation. Representative flow images were shown. Scale bar, 7 μM. (**B**) Quantification was determined by the manufacturer's software. These experiments were repeated at least three times.

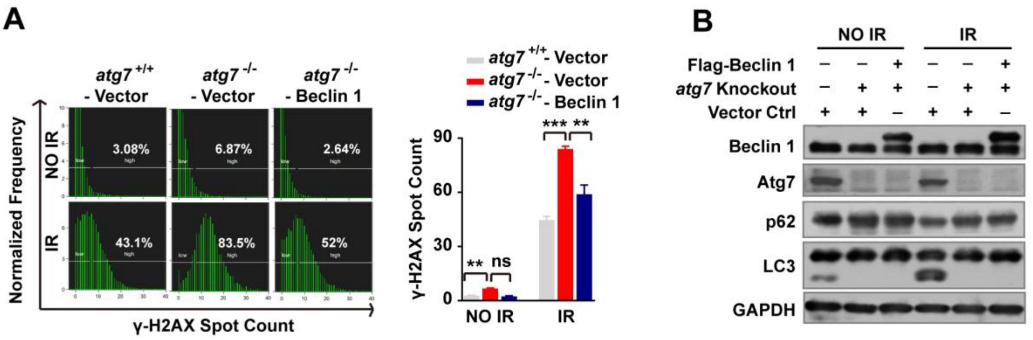

Figure S7. Overexpression of Beclin1 alleviates DNA damage repair but does not restore the autophagy response in autophagy-defective cells. (**a**) Image flow cytometric analysis of γ-H2AX spot counts in Atg7-null K562 cells transfected with or without beclin1 full-length plasmids. All bars show mean values ± SEM quantitated from ⩾3 independent experiments. *, $p<0.05$; **, $p<0.01$; ***, $p<0.001$. (**b**) Western blot analysis of Beclin1 overexpression experiments in which Atg7 was deleted. LC3 conversion and p62 protein level were analyzed in Atg7 knockout K562 cells. These experiments were repeated at least three times.

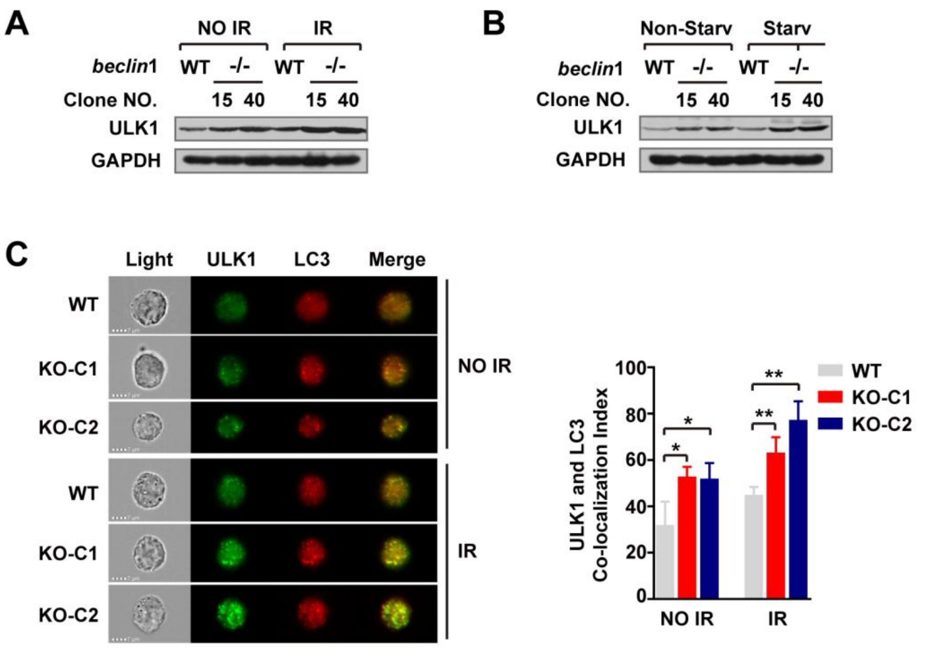

Figure S8. Beclin1 deletion leads to upregulation and focus formation of ULK1. (**A**) Immunoblotting detection to analyze the ULK1 in wild-type and beclin1 knockout Hela cells treated with or without 6 Gy ionizing irradiation, or (**B**) with or without starvation (HBSS). (**C**) ULK1 and LC3 co-localization was analyzaed using image flow cytometry 1h irradiation. The co-localization efficient was determined using the manufacturer's software (right) which is based on the similarity between two pixels, and representative flow images are shown (left).
